# Alveolar macrophages from EVALI patients and e-cigarette users: a story of shifting phenotype

**DOI:** 10.1186/s12931-023-02455-w

**Published:** 2023-06-17

**Authors:** Kristi J. Warren, Emily M. Beck, Sean J. Callahan, My N. Helms, Elizabeth Middleton, Sean Maddock, Jason R. Carr, Dixie Harris, Denitza P. Blagev, Michael J. Lanspa, Samuel M. Brown, Robert Paine

**Affiliations:** 1 Department of Internal Medicine, Division of Pulmonary & Critical Care Medicine, University of Utah, Salt Lake City, UT 84132 USA; 2 George E. Wahlen VA Medical Center, 500 Foothill Dr, Salt Lake City, UT 84148 USA; 3 Division of Pulmonary and Critical Care Medicine, National Jewish Health, Denver, CO 80206 USA; 4 Intermountain Healthcare, Department of Pulmonary & Critical Care Medicine, Murray, UT 84107 USA

**Keywords:** E-cigarette, Or vaping, Product use-associated lung injury (EVALI), Bronchoalveolar lavage (BAL), Alveolar macrophages (AM)

## Abstract

Exposure to e-cigarette vapors alters important biologic processes including phagocytosis, lipid metabolism, and cytokine activity in the airways and alveolar spaces. Little is known about the biologic mechanisms underpinning the conversion to e-cigarette, or vaping, product use-associated lung injury (EVALI) from normal e-cigarette use in otherwise healthy individuals. We compared cell populations and inflammatory immune populations from bronchoalveolar lavage fluid in individuals with EVALI to e-cigarette users without respiratory disease and healthy controls and found that e-cigarette users with EVALI demonstrate a neutrophilic inflammation with alveolar macrophages skewed towards inflammatory (M1) phenotype and cytokine profile. Comparatively, e-cigarette users without EVALI demonstrate lower inflammatory cytokine production and express features associated with a reparative (M2) phenotype. These data indicate macrophage-specific changes are occurring in e-cigarette users who develop EVALI.

## Introduction

E-cigarette, or vaping, product use-associated lung injury (EVALI) is an acute respiratory illness that inflicts substantial morbidity and mortality [1, 2]. The United States experienced a major outbreak of EVALI in 2019 predominantly in association with vitamin E acetate (VEA) adulteration of THC containing e-cigarettes [3]. This outbreak led to the recognition of EVALI and to thousands of illnesses and over sixty confirmed deaths [4]. Its clinical features include an acute inflammatory state associated with extensive ground glass opacities, neutrophilic alveolitis and increased numbers of lipid-laden macrophages (LLM) in bronchoalveolar lavage (BAL). Many EVALI patients have a rapid and dramatic improvement with corticosteroid treatment [2, 4]. Unlike lung injury caused by traditional cigarette use, EVALI develops rapidly in a young (< 35 years of age) healthy population [5].

At present, the pathobiology of EVALI is poorly characterized. It is probable there are multiple cellular contributors to its development [6], but the alveolar macrophage (AM) is likely to play a central role. Health agencies identified vitamin E acetate (VEA), a diluent often used in tetrahydrocannabinol (THC) e-cigarette liquids, as the causative agent leading to EVALI [7]. Subsequent analyses supported this association, as investigators demonstrated LLM and markers of inflammation in mice exposed to vaporized VEA that was not seen in mice exposed to the nicotine-containing JUUL™ (a brand of e-cigarettes) aerosol [7]. Interestingly, even during the initial EVALI outbreak, a small but significant minority of patients had no preceding exposure to THC [8]. Federal authorities removed much of the illicit THC from circulation later in 2019, resulting in a marked reduction in case counts [3, 5, 9–11]. In the years since the recognition of VEA as a major driver of the 2019 EVALI outbreak and through the COVID-19 pandemic, the number of patients with EVALI has decreased; nevertheless a steady number of patients continue to present with EVALI [12, 13]. Therefore, it remains unclear whether EVALI occurs because of a single vaping constituent or if it is a multifactorial disorder involving host-specific conditions (i.e. genetic predisposition), perhaps in combination with dose effects of vaping products.

Alveolar macrophages (AM) are lung resident cells that play a crucial role in host defense and repair in the lung. They have important roles in clearing pathogens and as sentinel cells that secrete inflammatory and anti-inflammatory mediators to recruit and activate other immune and inflammatory cells, including circulating mononuclear cells and neutrophils. Under normal circumstances, their activity is carefully regulated to optimize host defense while preventing impaired gas exchange and, ultimately, acute lung injury. Macrophages demonstrate a broad range of phenotypic characteristics, imperfectly captured along a spectrum characterized as M1 (classically activated/inflammatory) and M2 (alternatively activated/reparative) phenotypes [14]. Resident AM in the healthy lung strike a balance in which they express a mixture of M1 and M2 features [15]. In the setting of acute insults such as pneumonia, AM become fully activated, expressing abundant early response cytokines (TNF-α, IL-1β) and chemokines (IL-8 and CCL2), taking on an M1 predominant phenotype [16]. In addition to activation of resident AM during acute injury, circulating mononuclear phagocytes are recruited to the lung and differentiate into tissue (alveolar) macrophages [17]. In later stages, macrophages express M2 characteristics, such as expression of the reparative cytokine (IL-10) and chemokine (CCL22), allowing for resolution of the inflammatory response [18].

The consistent evidence of severe alveolar inflammation and morphologic changes found in AM of EVALI patients suggest that AM are a key target for investigation. We hypothesized that a localized immune response induces alterations in macrophage activity, which results in an exuberant inflammatory response in e-Cig users who develop EVALI. Here, we postulate that EVALI is a consequence of an exuberant inflammatory response to vaped substances by AM, resulting in recruitment and activation of neutrophils and recruitment of circulating mononuclear phagocytes, which contribute to the development of extensive alveolar exudates and respiratory failure.

## Methods

### Human subjects

Research subjects were recruited from the University of Utah and Intermountain Healthcare medical systems under IRB-approved protocols and provided informed consent. Eligible participants were between the ages of 18–50, had no history of chronic lung disease, and no recent respiratory symptoms/illness in the preceding four weeks. All EVALI subjects were hospitalized and met the CDC definition of “confirmed” or “probable” EVALI [19]. EVALI subjects were identified for enrollment by inpatient treating clinicians and recruited by study coordinators; none required mechanical ventilation or received corticosteroids prior to bronchoscopy. Subjects with EVALI were excluded if bronchoscopy presented excess risk for intubation by the treating clinician. Healthy subjects (healthy) and chronic users of e-cigarettes without EVALI (e-Cig controls) self-referred for enrollment. Healthy subjects reported no current or past use of cigarettes or vaping. E-cigarette users without EVALI self-reported current vaping every day, or most days, with no current cigarette use (< 100 traditional cigarettes in their lifetime and quit ≥ 1 year prior to enrollment). All healthy volunteers and three of the e-Cig subjects were recruited for a prior bronchoscopy study that was not published; BAL procedure and sampling protocols were identical across groups.

### Bronchoscopy and BAL collection

All subjects underwent bronchoscopy with BAL under moderate sedation. In addition to BAL, a standard e-cigarette exposure history was completed for subjects who were vaping. We obtained BAL fluid from healthy volunteers, e-Cig controls, and subjects hospitalized with EVALI. Each BAL was performed using a standardized protocol from the SPIROMICS Bronchoscopy Substudy and in line with previously published research bronchoscopy protocols [20, 21]. Briefly, subjects were allowed nothing by mouth for at least four hours prior to the procedure. Topical anesthesia was achieved using 1% lidocaine and moderate sedation was administered per hospital protocol. Subjects were continuously monitored with pulse oximetry, sphygmomanometry, and 3-lead ECG throughout the procedure. BAL was performed in the right middle lobe and lingula. Serial aliquots were instilled up to a total volume not to exceed 150 mL in each lung or 300 mL total. All samples were pooled, immediately stored, and transported on ice, and placed in a 4 °C refrigerator for short term (< 24 h) storage until further processing was completed.

### BAL cytospin preparation and quantitation

**Fig. 1:**
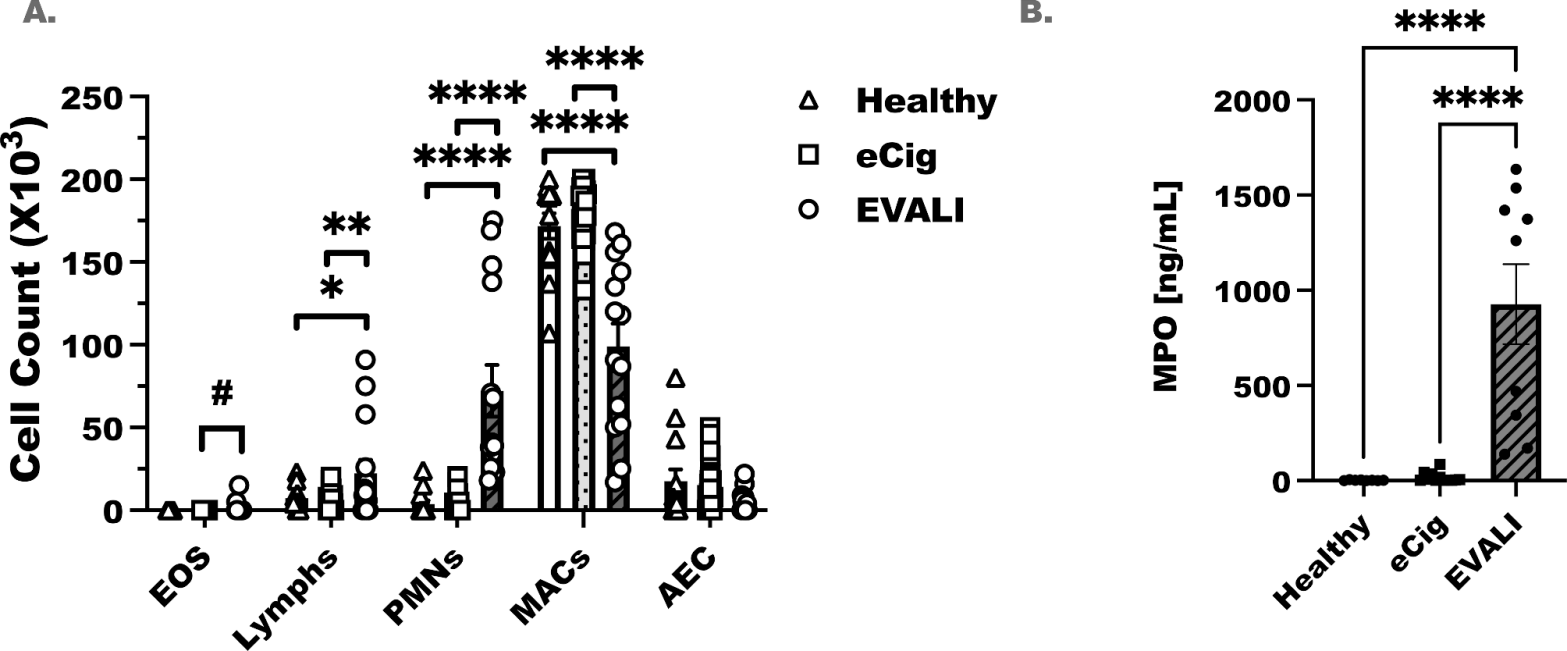
Increased neutrophils and MPO are detected in EVALI subjects. Healthy and e-cigarette controls were recruited as controls for EVALI subjects that had been admitted to the hospital for acute lung injury. Bronchoalveolar lavage fluids were collected following standard SPIROMICS procedures and cellular content was separated from supernatant. (**A**) Cytospins were prepared from the separate cells and eosinophils (EOS), lymphocytes (Lymphs), neutrophils (PMNs), Macrophages (MACs) and airway epithelial cells were quantified. At least 20–50 cells were counted field, 4–6 fields were counted per slide. Results as displayed as mean ± SEM. For healthy controls n = 7, e-cigarette controls (e-Cig) n = 13, and EVALI subjects n = 10. Statistical significance was determined by ordinary one-way ANOVA followed by Dunnett’s multiple comparison post-test to determine between groups differences

BAL cell counts were determined and 2,000–4,000 cells were diluted in 500 µL of sterile saline. 250 µL were applied to glass slides using the Thermo Scientific Cytospin 4 centrifuge; 2 slides were prepared for each study participant. Cells were applied to slides using the slide adaptors and filters (Biorad) by gentle centrifugation at 100 X g for 10 min at room temperature. Slides were dried at room temperature for 5 min, then treated with cold methanol for 5 min, before staining with Giemsa stain (Sigma) according to the manufacturers protocol, also described in Misharin et al. (2017) [22]. All slides were examined under an inverted light microscope. Eosinophils (Eos), neutrophils (PMNs), lymphocytes (Lymphs), macrophages (Macs) and ciliated airway epithelial cells were easily differentiated. 20–50 cells were quantified per field, 4–6 fields were assessed per subject. Finally, cell numbers were normalized to 200 cells per cell type and graphed as mean cell count per standard error of the mean (SEM). These data are shown in Fig. 1.

### Flow cytometry

**Fig. 2:**
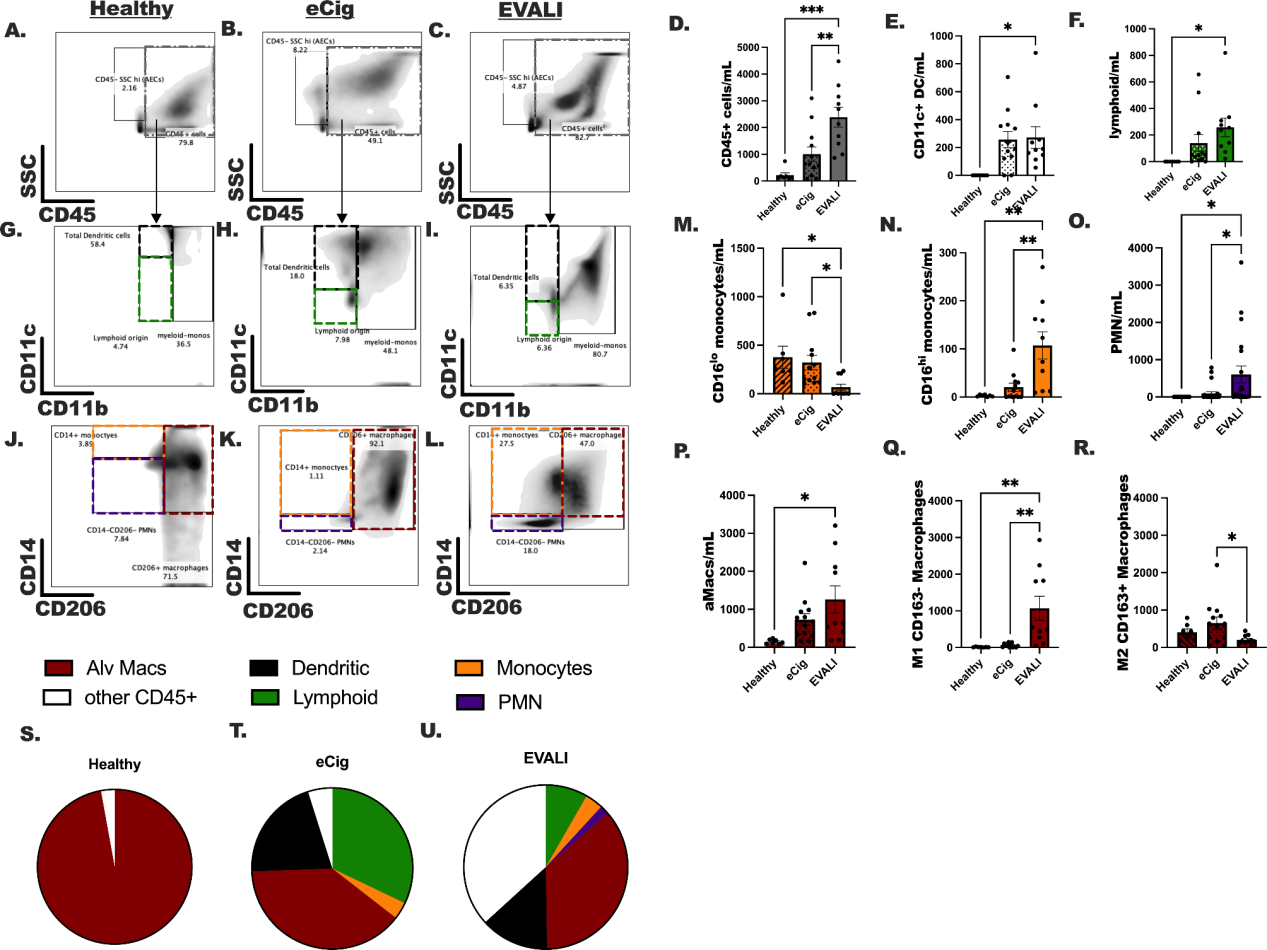
Composition of immune lineage cells differ in EVALI compared to otherwise healthy e-cigarette controls. Flow cytometric analysis was completed using a macrophage specific flow panel that included a viability dye, and antibodies specific for CD45, CD11c, CD11b, CD14, CD16, CD206, CD64 and CD163. (**A**)-(**C**) Total immune cells were detected as CD45 + cells in three groups healthy participants (**A**; healthy), e-cigarette controls (**B**; e-Cig), and EVALI subjects (**C**; EVALI). (**D**) The counts of CD45 + cells were normalized per mL of returned lavage fluid and shown as count/mL. (**G**-**I**) gating schematic and counts of (**E**) dendritic cells and (**F**) lymphocytes per mL of returned BAL fluid. (**M**-**O**) quantitation for (**M**) CD16^lo^ and (**N**) CD16^high^ inflammatory monocytes and (**O**) neutrophils and (**J**-**L**) the representative flow gating for each group. (**J**-**L**) shows the representative gating for alveolar macrophages, populations are contained in the dark red box. The quantitation of (**P**) total alveolar macrophages (aMacs), (**Q**) CD163- M1 macrophages and (**R**) CD163 + M2 macrophages. The composition of total BAL of each of the above cell populations are displayed in **S**-**U** for each group. Statistical significance was determined using an ordinary One-Way ANOVA with Dunnett’s post-test to determine between groups differences; *indicates p < 0.05, ** indicates p < 0.01, *** indicates p < 0.001. Sample size is n = 7 for healthy controls, n = 13 for e-Cig controls and n = 10 for EVALI subjects

Cells were separated from supernatants by gentle centrifugation at 300 X g for 10 min. Total BAL cells were counted using trypan blue exclusion. 1–3 × 10^6^ cells were added into 5 mL polystyrene tubes (Falcon) containing Zombie Aqua (Biolegend; cat# 77,143). The following anti-human antibodies were acquired from Biolegend: APC-Fire 750 HLA-DR (clone: I.243; cat#307,657), CD14 conjugated to Brilliant Violet 711 (clone: M5E2; cat# 301,837), CD163 conjugated to PE-Cy7 (clone: GHI/61; cat#333,613), CD64 conjugated to Brilliant Violet 605 (clone: 10.1; cat# 305,033), CD11b conjugated to Brilliant Violet 750 (clone: M1/70; cat# 101,267). Anti-Human CD11c conjugated to AF700 was acquired from Invitrogen (clone: 3.9; cat# 56-0116-41). The remaining anti-human antibodies were purchased from BD Biosciences; anti-human CD16 conjugated to BUV 496 (clone: 3G8; cat# 612,945), anti-human CD45 conjugated to BUV 805 (clone: HI30, cat# 612,891) and anti-human CD206 conjugated to BUV 395 (clone: 19.2; cat# 740,309). Compensation was completed using UltraComp eBeads from Invitrogen (cat# 01-2222-42), Zombie aqua only stained and unstained cellular events were collected for each sample including the healthy, e-Cig and EVALI groups to properly set flow cytometric gating for data acquisition on the Cytek Aurora. Individual samples were further analyzed using FlowJo, v.10 software, using unstained cellular events from each study subject to set flow gating (shown in Fig. 2).

### ELISA

A 1 milliliter aliquot of bronchoalveolar lavage fluid was pre-cleared by centrifugation (500 x g for 10 min) prior to analysis by ELISA. All ELISA were performed according to the manufacturer’s instructions without modifications. A BCA (cat# 71285-3) protein assay was purchased from Millipore. Human IL-6 (cat# DY206), IL-8 (DY208), RAGE (DY1145), and CRP (Cat# QK1707) ELISA kits were acquired from R&D Systems (Minneapolis, MN). Human serum amyloid A, or SAA (cat# KHA0011), MPO (BMS2038INST), and sICAM ELISA kits (cat# BMS201) were acquired from Invitrogen (Vienna, Austria). Absorbance readings for the standard curve and experimental samples were detected at 450 nm with 570 nM correction on the SpectraMax M3 from Molecular Devices. Concentrations of detected proteins were extrapolated from standard curve and displayed as mean +/- the standard error of the mean in Fig. 2.

### Statistical analysis

Data are presented as the mean ± standard error of mean (SEM). Statistics were performed using one-way analysis of variance (ANOVA) after confirmation that all data were normally distributedusing D’Agostino & Pearson testing. Tukey’s multiple comparison, post-tests were employed to compare differences across the three groups. All statistical analysis were completed on GraphPad Prism (version 9). In all analyses, P values less than 0.05 were considered statistically significant.

**Table 1:** Study Participant Characteristics

	Healthy	E-Cig	EVALI
**Number**	7	13	10
**Age (median)**	28	23	22
**Reported Sex**			
Female (%)	2 (29)	7 (54)	5 (50)
Male (%)	5 (71)	6 (46)	5 (50)
**Race**			
Caucasian (%)	3 (43)	9 (69)	8 (80)
American Indian or Alaskan Native (%)	0 (0)	0 (0)	1 (10)
Native Hawaiian or Other Pacific Islander (%)	0 (0)	1 (8)	0 (0)
Other (%)	0 (0)	1 (8)	0 (0)
Undisclosed/Refused (%)	4 (57)	2 (15)	1 (10)
**Ethnicity**			
Non-Hispanic (%)	3 (43)	10 (77)	7 (70)
Hispanic (%)	0 (0)	1 (8)	2 (20)
Undisclosed/Refused (%)	4 (57)	2 (15)	1 (10)
**Years Vaped (median)**	n/a	3	2.5
**Product Vaped**			
Nicotine (%)	n/a	11 (85)	6 (60)
THC/CBD (%)	n/a	4 (31)	10 (100)
Dual-Use (%)	n/a	2 (15)	6 (60)*
**Number Puffs Per Day**			
<10 Puffs Per Day (%)	n/a	5 (50)**	2 (20)
>10 Puffs Per Day (%)	n/a	5 (50)**	8 (80)

*1 subject denied THC use but urine testing was positive for THC metabolites; this subject was re-classified as a dual-user

** Puffs/day not collected in 3 subjects

## Results

Participant characteristics are summarized in Table 1. In general, subjects were young (< 35 years of age), Caucasian, and non-Hispanic. We enrolled 10 EVALI subjects, 13 e-Cig controls, and 7 healthy controls. Nine (90%) EVALI subjects were hospitalized for acute hypoxemic respiratory failure (requiring supplemental oxygen) and received antibiotics covering organisms that contribute to community acquired bacterial pneumonia; one (10%) subject was hospitalized but did not require supplemental oxygen. The median time from hospital admission to bronchoscopy was one day. Seven (70%) of EVALI subjects had a “confirmed” diagnosis by CDC criteria. The three remaining subjects met “probable” EVALI criteria: two tested positive for rhinovirus/enterovirus on nasal viral PCR, and the other had an incomplete rheumatologic workup. The treating clinicians and research team classified these subjects as “probable” given that the clinical history was more consistent with EVALI than competing diagnoses; the two subjects with positive nasal swabs had negative viral panels on BAL and no medical history predisposing to respiratory failure from these viruses. Only one subject required intensive care unit (ICU) admission for their hypoxemia; the remaining subjects in the EVALI group who required supplemental oxygen support by simple nasal cannula were admitted to the medicine wards.

### Increased numbers of neutrophils are present in BAL of EVALI subjects

BAL cytospins were examined as a crude means of understanding general immune mediated lung inflammation. Numbers of eosinophils (Eos), lymphocytes (Lymphs), neutrophils (PMNs), macrophages (Macs) and airway epithelial cells were determined in EVALI subjects, healthy controls, e-Cig controls (Fig. 1A). Lymphocytes and neutrophils were increased in EVALI in comparison to both healthy (p < 0.05 lymphocytes and p < 0.0001 for PMN) and e-Cig controls (p < 0.01 for lymphocytes and p < 0.0001 for PMN). Healthy and e-Cig controls maintained higher numbers of macrophages in their BAL as compared to EVALI (p < 0.0001). Although two EVALI subjects had eosinophils detected in BAL, this difference did not achieve statistical significance (p = 0.0752). Only EVALI subjects had increased amounts of myeloperoxidase (MPO) compared to healthy (p < 0.0001) and e-Cig controls (p < 0.0001) (Fig. 1B). Finally, although readily visualized and quantified, there were no statistical differences in the numbers of alveolar epithelial cells between groups (EVALI to healthy comparison p = 0.113).

### Cellular composition of BAL differs according to EVALI and e-Cig group status

The concentration of CD45 + cells per mL were increased in EVALI compared to e-Cig (p < 0.01) and healthy controls (p < 0.001) (Fig. 2A-D). CD11c^+^CD11b^−^ dendritic cells (DC) were detected with SSC^lo^ FSC^+^CD11b^−^CD11c^−^CD206^−^CD14^−^ cells, that are likely lymphoid origin cell populations (Fig. 2E, F, G-I). DC and lymphoid origin cells were only statistically different in the EVALI subjects compared to healthy controls (p < 0.05); there were no differences between e-Cig subjects and EVALI subjects for either of these cell populations. Total monocytes were detected, and discriminated as CD16 ^lo^ (Fig. 2M) and CD16 ^hi^ (Fig. 2N) monocytes, to distinguish classical monocytes from inflammatory monocytes, respectively. EVALI subjects had a higher CD16 ^hi^ population of monocytes when comparing to healthy (p < 0.05) and e-Cig controls (p < 0.05), and healthy and e-Cig controls had a higher concentration of CD16 ^lo^ classical monocytes as compared to EVALI subjects (p < 0.01). As with the cytospin data, we were able to confirm an increased accumulation of neutrophils in the BAL fluid of EVALI subjects compared to both healthy controls (p < 0.05) and e-Cig controls (p < 0.05) (Fig. 2O). Finally, alveolar macrophages were examined (Fig. 2P) and discriminated along an M1-M2 spectrum based on CD163 and HLA-DR staining: M1-skewed macrophages were determined as HLA-DR ^hi^ CD163^−^ (Fig. 2Q); while M2-skewed macrophages were also HLA-DR^hi^ they were also CD163^+^ (Fig. 2R). Total macrophages were increased in EVALI subjects compared to healthy (p < 0.05). EVALI subjects had more CD163^−^ macrophages in comparison to healthy (p < 0.01) and e-Cig controls (p < 0.01). Contrastingly, e-Cig controls had more CD163^+^ macrophages as compared to EVALI subjects (p < 0.05), and healthy controls had comparable numbers of CD163^+^ macrophages to the e-Cig controls. Finally, the composition of BAL cell populations were summarized in Fig. 2S and U.

### Markers of inflammation and airway leak are increased in BAL from EVALI subjects in comparison to e-Cig and healthy controls

**Fig. 3:**
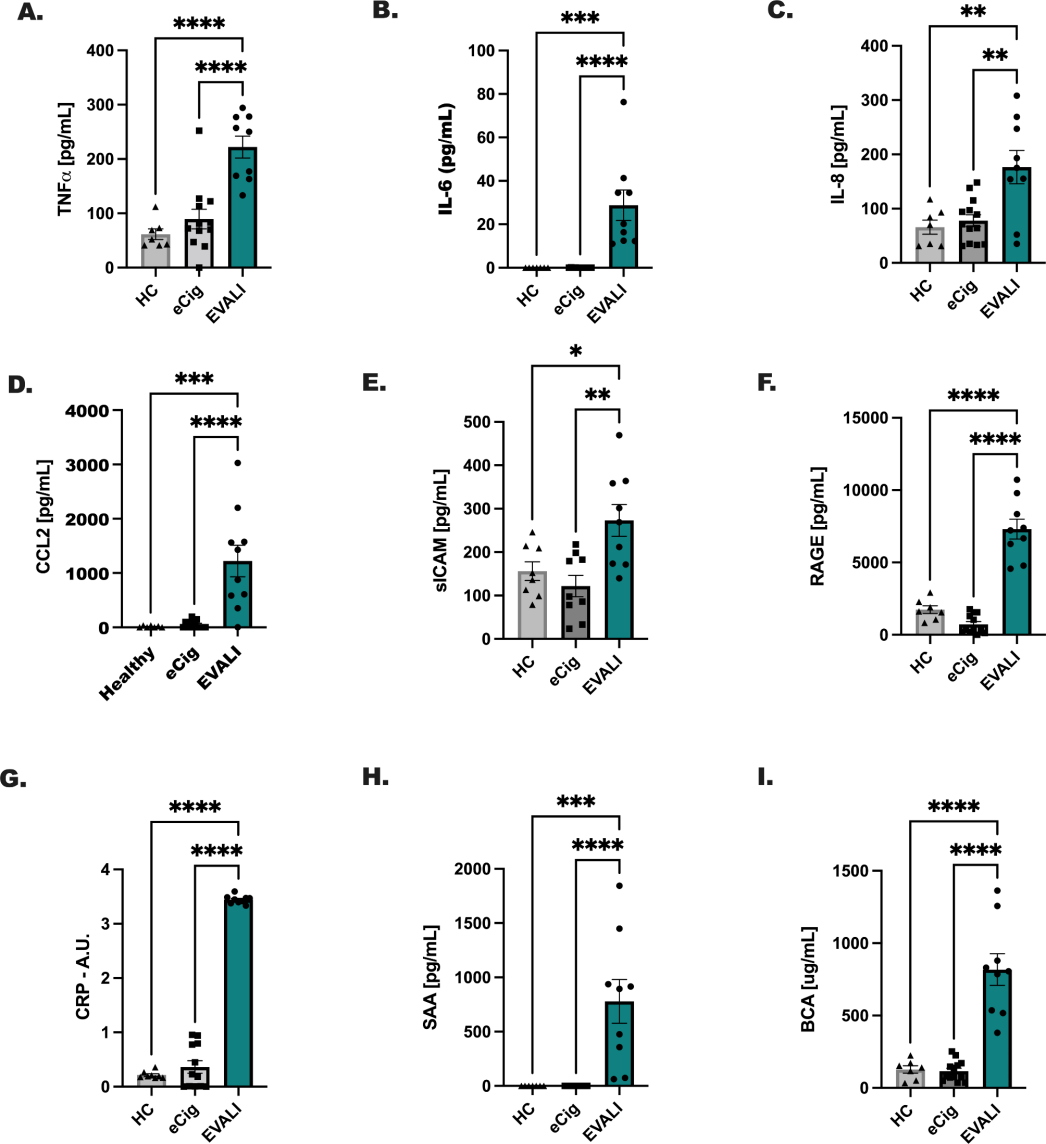
Markers of inflammation and airway leak are increased in EVALI subjects compared to e-cigarette and healthy controls. As above, BAL was precleared of cellular content and analyzed for markers of inflammation; (**A**) TNFα, (**B**) IL-6, (**C**) IL-8, (**D**) CCL2, (**E**) soluble ICAM and (**F**) RAGE by ELISA. Markers of airway leak were also detected; (**G**) C reactive protein (CRP), (**H**) serum amyloid A (SAA), and total protein (BCA; µg/mL). Statistical significance was determined using an ordinary One-Way ANOVA with Dunnett’s post-test to determine between groups differences; *indicates p < 0.05, ** indicates p < 0.01, *** indicates p < 0.001, **** indicates p < 0.0001. Sample size is n = 7 for healthy controls, n = 13 for e-Cig controls and n = 10 for EVALI subjects

Prototypical markers of inflammation, including TNFα, IL-6, and the chemokines, IL-8 and CCL2, were increased in EVALI patients compared to healthy (p < 0.01) and e-Cig controls (p < 0.01)(Fig. 3A-D**)**. Additional markers of lung pathogenesis, sICAM, RAGE, total protein, serum amyloid A and C-reactive protein, were all detected at higher levels in BAL fluid from EVALI patients compared to healthy (p < 0.01) and e-Cig (p < 0.01)(Fig. 3E-I). Of note, as with all the results thus far, healthy controls and e-Cig controls were similar and had virtually no markers of inflammation in their BAL fluid.

## Discussion

Understanding the biologic underpinnings of EVALI is required to aid in the prevention, diagnosis, and treatment of this novel syndrome. We aimed to differentiate AM phenotypes in routine e-cigarette use from EVALI. Macrophage differentiation along a spectrum towards an M1 phenotype is a finding seen in various forms of acute lung injury and acute respiratory distress syndrome (ALI/ARDS) in both murine and human studies [16, 23, 24]. In the case of ALI, macrophages are activated towards the M1 phenotype, with release of pro-inflammatory cytokines, such as tumor necrosis factor-alpha (TNF-α), interleukin-1 beta (IL-1β), and interleukin-6 (IL-6), and the production of reactive oxygen and nitrogen species (ROS and RNS, respectively). While this response is critical for the initiation of the immune response to ALI, it is also nonspecific and can be triggered by a variety of insults, including viral infections, bacterial infections, and chemical exposure. Here, we demonstrate that AM from individuals with EVALI display features of an inflammatory, or M1-type, phenotype. In contrast, AM from e-Cig controls without EVALI express M2-skewed features, similar to AM from healthy controls. Neutrophilic inflammation, airway leak, and elevated IL-8 and CCL2 fit well with the M1 macrophage phenotype in the EVALI subjects. Neutrophilic infiltration in the airways contributes to the pathogenesis of COPD and other inflammatory lung diseases [25, 26]. Electronic-cigarette controls and healthy controls demonstrate comparable levels of CD163^+^ (M2-skewed) macrophages with no inflammatory cytokine profile detected. The CD163 marker was elevated on macrophages of e-Cig controls compared to EVALI subjects, however, indicating that macrophages from e-Cig controls maintain a relatively anti-inflammatory state. Together the data suggest that interventions to maintain AM in a normal, more anti-inflammatory state (associated with CD163 expression) might prevent or improve outcomes associated with EVALI. This line of reasoning regarding CD163 on AM of course requires more exploration before becoming clinically relevant.

The specific components of the vaping mixture used in e-cigarette devices likely contribute to the development of EVALI [27, 28]. All EVALI subjects used THC products, while only 30% (4/13) of the e-Cig group reported THC use. In the 2019 EVALI outbreak, > 85% of patients had presumed THC exposure and 94% had VEA detected in BAL fluid [3]. Mice exposed to aerosolized VEA developed diffuse lung injury with a neutrophilic and monocytic inflammatory infiltrate as well as an increase in lung water and BAL protein levels [7]. The relative contributions of different vaping constituents for human EVALI is not yet clear but is the subject of active ongoing investigation.

Chronic e-cigarette use may predispose users to developing clinical disease by increasing susceptibility to respiratory infection [29]. In a study where mice were exposed to e-cigarette aerosols with and without nicotine, AM demonstrated intracytoplasmic inclusions and M2 markers as compared to air-exposed animals [30]. However, mice exposed to e-cigarette aerosols had an excessive inflammatory response to the influenza A viral infection which resulted in increased levels of IFN-γ and TNFα. While histologic changes also demonstrated increased inflammation and edema, those animals exposed to e-cigarette vapors had decreased survival suggesting this hyperactive immune response was mounted due to vaping exposure that led to detrimental immune pathology in the delicate lung [30]; the cascade suggests e-cigarette aerosols prime the subject for an excessive inflammatory response to viral infection. This was similarly reported in an in vitro model using e-cigarette extracts on human small airway epithelial cells [31]. In contrast, in our human study we did not see increased inflammatory proteins (e.g. TNFα, MPO) in e-Cig controls. Only two of the EVALI subject tested positive for rhinoviral infection. Other studies have reported normally non-noxious pathogens in EVALI subjects [8]. Whether e-Cig use primes the lung for excess inflammatory response to these normally benign pathogens leading to EVALI in some individuals requires additional investigation.

Our results add to prior studies examining the lungs’ immune responses to e-cigarette use. We did not evaluate pulmonary physiologic responses to e-cigarette use, as has been done in prior studies, which demonstrated alterations in heart rate, blood pressure, arterial stiffness, and oxygen tension [32–34] in subjects vaping nicotine-based solutions, but we did examine the inflammatory profile and immune response like prior investigators and arrived at different findings. As opposed to previous studies [35–37], we did not find increased BAL neutrophil or lymphocyte cellularity in our regular e-Cig users, though they were found in higher degrees in subjects with EVALI. Similarly, our EVALI subjects demonstrated increased BAL inflammatory markers like TNFα and IL-8, as opposed to the e-Cig group which largely resembled healthy controls. This finding differs from prior studies of the lungs’ response to e-liquids with nicotine, which have found abnormal inflammatory features [34, 35, 38] and evidence of direct airway damage [39], A limitation of our observational study is that we did not standardize exposures – such as the flavors permitted, nicotine/THC strength, frequency of use, or the time interval between the time of last e-cigarette use and the time of research bronchoscopy – in our e-Cig group; prior studies that demonstrated immune or inflammatory changes specifically accounted for these. It is thus feasible the negative results witnessed in the e-Cig group could be a result this variability, but further research is needed to confirm or refute this possibility.

Generalizing findings from animal studies to human pathogenesis of disease is challenging. As our findings demonstrate, the alveolar cellular composition in e-Cig controls resembles healthy controls and most e-Cig controls do not go on to develop EVALI. It may be that the pathogenesis of EVALI is that of a multi-hit model where chronic exposure to e-cigarette vapors in the setting of differences in host characteristics, inadequately understood differences in e-cigarette constituents (such as exposure to VEA), and/or exposure to common pathogens, result in clinical disease.

There are several limitations to our study: first, there are no accepted standards on the quantity and quality of e-cigarette constitutes, devices, or post-market modifications. We did not analyze the composition of e-cigarette vapors or e-liquid composition or assess the generation (1st – 4th ) of e-cigarette devices; recent reports have suggested that the later generation e-cigarette devices mitigate the progression to EVALI [40]. Post-marketing modifications to achieve higher temperatures or higher levels of THC or nicotine were not accounted for and we did not routinely confirm THC/nicotine metabolites present in our subjects (THC and nicotine use were self-reported). Only two of the ten EVALI subjects were tested for THC through routine clinical testing (not conducted as part of this study); both tested positive. Second, we did not examine the distinct morphologic features of AM obtained from EVALI subjects versus e-Cig controls to determine whether lipid-packed vacuoles were present in EVALI subjects and not in e-Cig controls, or vice versa. Initial reports of EVALI identified the presence of lipid-laden macrophages (LLM) in EVALI patients. However, LLM are present in BAL fluid of smokers of traditional cigarettes and are also seen in various other conditions such as lipoid pneumonia, pulmonary alveolar proteinosis, gastroesophageal reflux, and aspiration syndromes and thus, are not specific to EVALI [29, 41–43]. Finally, unknown confounders may exist that could be important, including brand of vaping liquid, depth of inhalation, number of puffs/session, number of sessions/day, and time from last vaping session to symptom onset/hospital presentation; we received limited data from our subjects regarding this information.

An important strength of this study is the comparison of individuals with EVALI to e-Cig users without respiratory illness. While we examined several inflammatory proteins, we were unable to identify a specific biomarker that underpins the transition from health to EVALI. There may be additional biomarkers of exposure and e-cigarette effect not included in the analysis. Future studies should evaluate sequencing differences and differences in lipid homeostasis in the macrophages and airway cells. Other markers of lung integrity (i.e., surfactants), and functional analysis of AM (phagocytosis) are important to establish in future studies, as well. This report is the first study that specifically investigates a comprehensive lung immune cell profile in subjects with EVALI, and compare EVALI subjects to both healthy and chronic e-Cig controls.

In summary, we have demonstrated that EVALI induces a robust inflammatory response that is mediated at least in part by an inflammatory AM phenotype and that the AM in chronic e-Cig users more closely resembles healthy control subjects. Given these findings, we speculate that EVALI likely develops as part of a sequence of insults that culminate in exuberant inflammation and respiratory compromise.

## Data Availability

All data generated and analysed during this study are included in this published article.
